# Moving Beyond the Limits of Detection: The Past, the Present, and the Future of Diagnostic Imaging in Canine Osteoarthritis

**DOI:** 10.3389/fvets.2022.789898

**Published:** 2022-03-15

**Authors:** Gareth M. C. Jones, Andrew A. Pitsillides, Richard L. Meeson

**Affiliations:** ^1^Department of Clinical Science and Services, Royal Veterinary College, Hatfield, United Kingdom; ^2^Department of Comparative Biological Science, Royal Veterinary College, London, United Kingdom

**Keywords:** MRI, CT, radiography, osteoarthritis, dog, canine, degenerative joint disease, state of the art review

## Abstract

Osteoarthritis (OA) is the most common orthopedic condition in dogs, characterized as the chronic, painful end-point of a synovial joint with limited therapeutic options other than palliative pain control or surgical salvage. Since the 1970s, radiography has been the standard-of-care for the imaging diagnosis of OA, despite its known limitations. As newer technologies have been developed, the limits of detection have lowered, allowing for the identification of earlier stages of OA. Identification of OA at a stage where it is potentially reversible still remains elusive, however, yet there is hope that newer technologies may be able to close this gap. In this article, we review the changes in the imaging of canine OA over the past 50 years and give a speculative view on future innovations which may provide for earlier identification, with the ultimate goal of repositioning the limit of detection to cross the threshold of this potentially reversible disease.

## Introduction

Osteoarthritis (OA) is the most common orthopedic condition observed in dogs (1), with estimated clinical prevalence of ~2.5% (2, 3) that increases to 20% when evaluated *post-mortem* (4, 5). OA has major impacts on dog welfare due to its severity, long duration and requirement for chronic pain management (3). The impact is similar in humans, with the 2016 Osteoarthritis Research Society International (OARSI) white paper identifying OA as the cause of 2.4% of all years lived with a disability in humans, in which global prevalence of knee and hip OA approaches 5% (6).

Frustratingly, the earliest OA changes to the joint organ are currently undetectable, whereas the clinically recognizable OA syndrome causing pain and disability only develops toward the culmination of OA pathology, when there are limited to absent therapeutic options available. Currently treatments mainly involve palliative pain control or surgical salvage to remove the diseased joint entirely. Common diagnostic imaging approaches, and principally the use of radiography, have a critical role in OA diagnosis, however, earlier disease intervention is limited by the sensitivity of radiographic imaging and by its failure to achieve correlation between the types of OA changes identifiable and clinical joint function. There remains a need to identify changes earlier in the disease process, which has led to the utilization of newer imaging technologies such as magnetic resonance imaging (MRI) and computed tomography (CT) in both humans and dogs to try and close the gap between the onset of pathology and the limit of imaging detection.

**Figure 1:**
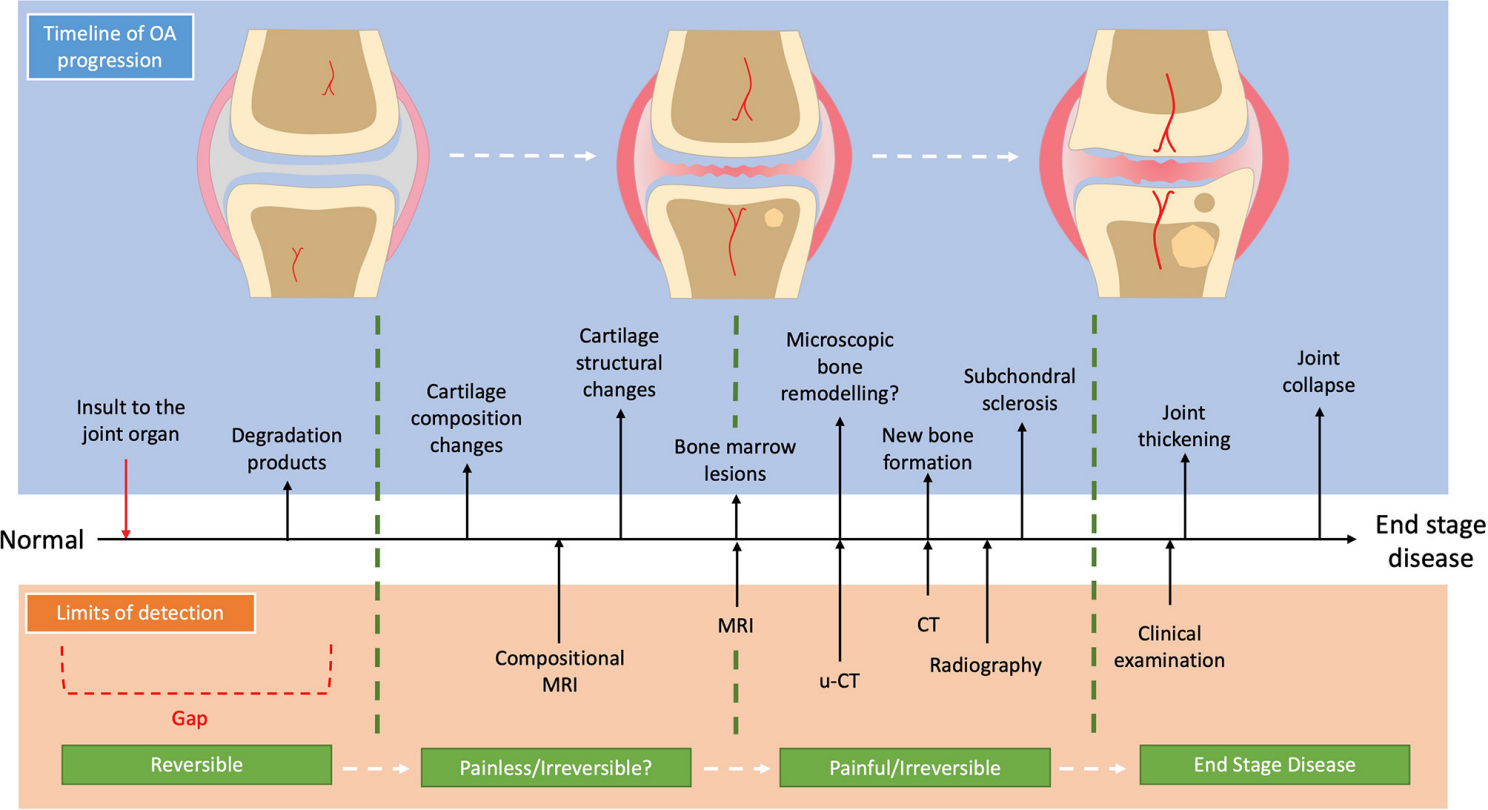
Schematic progression of OA and the current detection limits with diagnostic imaging.

Identifying and developing the correct diagnostic modality requires full awareness of OA aetiopathogenesis and trajectory within the context of distinct OA sub-divisions. OA is no longer considered to be only a simple failure of the articular cartilage but is characterized as a demise of the “joint organ” where there is increasing focus on the osteochondral unit (7) and its temporospatial changes during OA progression (7–9). One current view of the temporal sub-divisions of OA is that the composition of cartilage changes early in disease progression (10), which makes the cartilage more susceptible to load-induced damage leading to fibrillation of its surface. In response, the chondrocytes attempt to repair the cartilage by upregulating their activity with production of several pro-inflammatory mediators as well as matrix degradation products which promote extra-cellular matrix turnover (11). This increase in matrix turn-over becomes imbalanced favoring catabolic degradation, while the pro-inflammatory state stimulates a proliferative response in the synovium further potentiating the pro-inflammatory environment (12). In parallel to the cartilage changes, the subchondral bone responds with increased bone turnover which in the early stages leads to a more porous and thinned subchondral plate (13), with increased vascular invasion and the formation of bone-marrow lesions (BMLs) (14). As OA progresses to the later stages, the cartilage continues to be degraded with deeper lesions occurring. Within the cartilage, chondrocytes undergo either hypertrophy and clonal expansion or apoptosis (7). The subchondral bone continues to remodel with the subchondral bone plate becoming thicker and the trabecular bone becoming more sclerotic (15). Further bone remodeling occurs with the development of subchondral bone cysts as well as periarticular osteophytes (7). This view of the aetiopathogenesis of OA is summarized in Figure 1, accompanied by the current limits of detection of each imaging modality for OA.

This review will consider the past and current steps in the advancement of diagnostic imaging in canine OA, where the evolving development of technologies have progressively allowed the observer to identify earlier and earlier biomarkers of OA disease. Finally, this review will provide a speculative view of future innovations which may provide for identification of an earlier disease state, with the ultimate goal of the limit of detection crossing over the threshold of pathologically reversible disease.

## The Past and Present; Radiography

Prior to the 1970s, the diagnosis of dog OA was based solely on clinical evaluation. Clinicians made the provisional diagnosis based on the presence of joint pain, joint effusion, soft tissue thickening, and crepitus, without use of diagnostic imaging. Although this approach is most closely aligned with the patient, understanding of the pathology was limited to post-mortem specimens only, and a definitive diagnosis in the live patient was elusive. However, with the integration of radiography into veterinary practice in the 1970s, there was now a means to definitively diagnose OA before some of the more end-stage clinically appreciable changes appeared.

**Figure 2:**
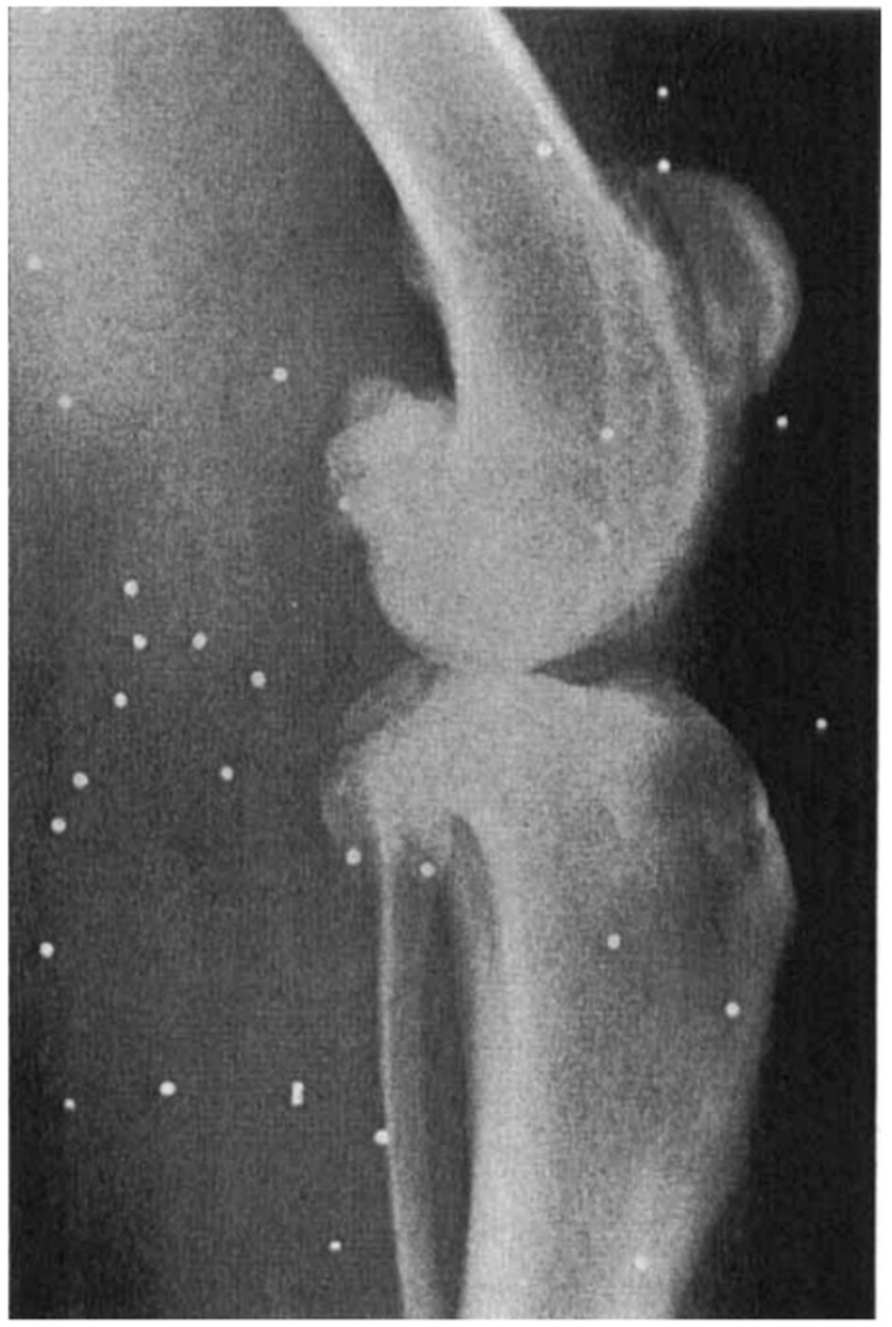
Lateral radiograph of an older clinically-lame dog in the non-weight bearing position, presented by Morgan in 1969. The radiograph shows evidence of periarticular osteophytosis, subchondral sclerosis and joint space narrowing. Morgan JP. Radiological Pathology and Diagnosis of Degenerative Joint Disease in the Stifle Joint of the Dog. J Small Anim Pract. 1969; 10:541–4. Reprinted with permission.

One of the first publications depicting the changes seen with spontaneous clinical OA in the dog, was published in the Journal of Small Animal Practice by J. P. Morgan in 1969 (16), although the radiographic changes had been described in textbooks and experimental canine models prior to this date. Morgan compared radiographs of the stifle joint in 12 young, clinically normal dogs, to those in 12 older, clinically lame dogs, in both a non-weight bearing and weight bearing position. The OA joints demonstrated radiographically identifiable periarticular osteophytes, subchondral sclerosis, joint space collapse, subluxation and rotation; some of these changes are shown in Figure 2.

**Figure 3:**
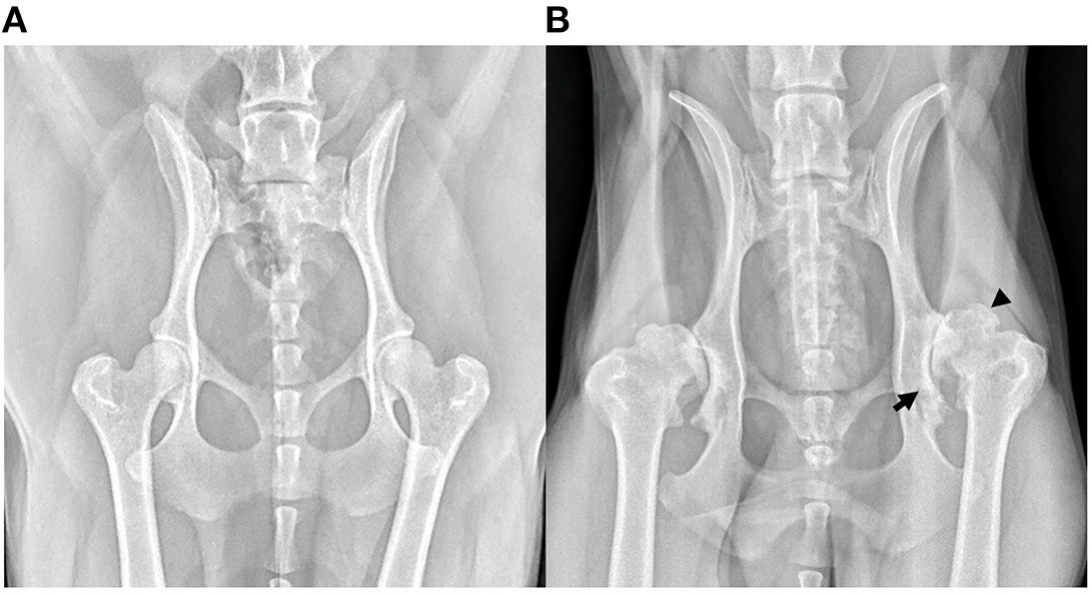
Ventrodorsal extended limb radiographs highlighting hip osteoarthritis (OA). **(A)** Radiograph of an adult dog with radiographically normal hips. **(B)** Radiograph of a middle-aged, male, large-breed dog with severe OA secondary to hip dysplasia. This radiograph shows evidence of advanced new bone formation (osteophytosis, black arrow head), sclerosis and remodeling of the acetabulum (black arrow).

**Figure 4:**
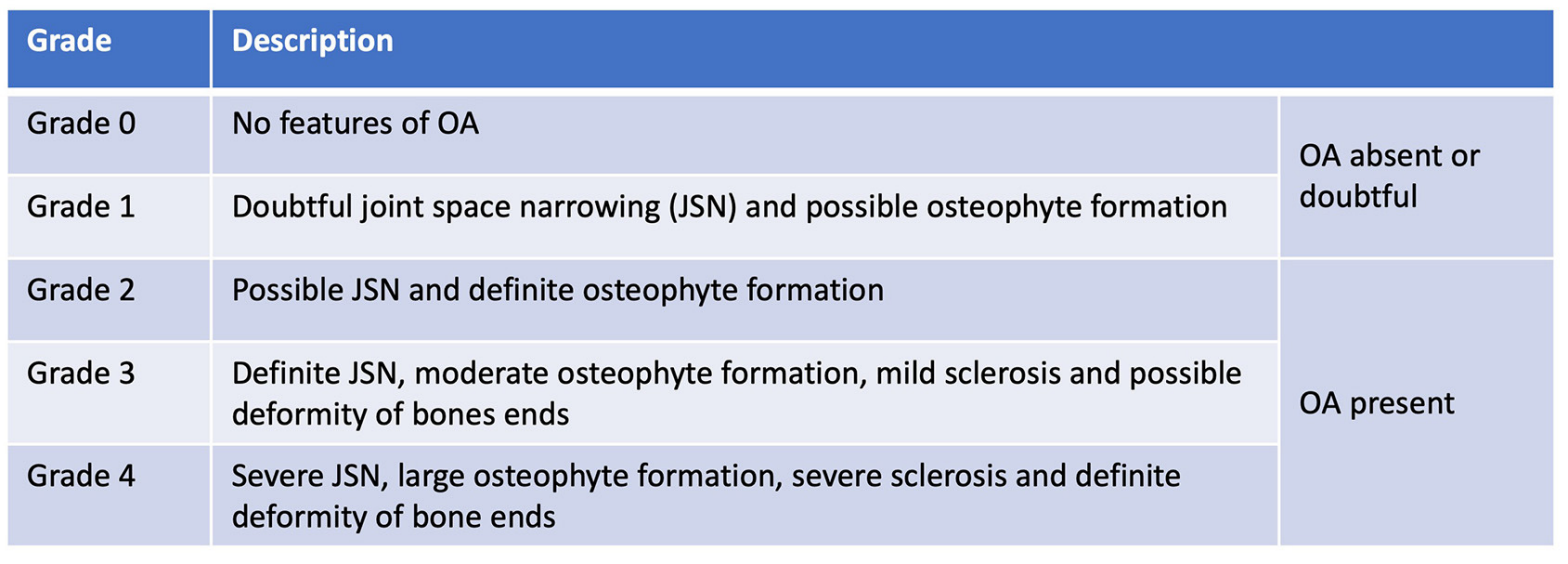
Kellgren Lawrence Scoring system, published in 1957. Kellgren JH, Lawrence JS. Radiological assessment of osteo-arthrosis. Ann Rheum Dis. 1957; 16(4):494–502.

Soon after Morgan's landmark publication, similar findings were described in other joints; first in a short case-series in 1970 assessing the changes in the stifle at post-mortem examination (17), then in larger cadaveric surveys of radiographic and pathological aspects of OA in the shoulder (18, 19), elbow (19, 20) and stifle (4). As radiography became more accessible in general practice, the Journal of Small Animal Practice published a series of articles titled ‘Radiological refreshers', including the radiological examination of stifle OA, in which the locations for osteophyte assessment were highlighted (21). Periarticular osteophytosis, subchondral sclerosis, joint swelling and effusion, joint remodeling, and to a lesser extent joint space narrowing (JSN) have since remained the key radiographic hallmarks in the diagnosis of OA in the dog (Figure 3). These radiographic markers have been used to produce various scoring systems (22–28), both in experimental canine models for human OA and in prospective spontaneous clinical veterinary studies. However, in comparison to human medicine, no single scoring system has been accepted as the standard-of-care in the diagnosis of canine OA with radiography. In comparison, despite its accepted shortcomings (29), the Kellgren-Lawrence (KL) scoring system has been the accepted standard-of-care for radiographic diagnosis for human OA since 1958, with grading based on descriptive definitions (Figure 4) (30, 31). Other systems have been developed, including the OARSI atlas assessment, first published in 1996 (32), then revised in 2007 (33), to attempt to address some of the shortcomings of the KL system. The main difference with the atlas assessment, is that radiographs are compared to a database of images to assess for ‘best-fit', as opposed to descriptive grades, in an attempt to reduce inter-observer variability. Although the acceptance of OA grading in human medicine is not without issue, it is clear however that the adoption of a standardized radiographic scoring of canine OA could have a positive impact on both diagnosis and monitoring. Furthermore, these systems could also improve the standardization and then subsequent reliability in prospective trials with disease modifying therapies for OA, for both veterinary clinical and potential *one medicine* orientated studies.

Despite its lack of sensitivity to early stages of OA detection, radiography remains the standard-of-care for OA diagnosis in dogs and humans being cheap, widely available, well established and safe. However, a further issue is that the severity of radiographic changes do not necessarily accurately correlate to clinical disease. This was noted by Olsson in 1971 in his comment that the diagnosis of OA should not be made on the presence of osteophytes alone, and secondly, that clinical signs are often unrelated to radiographic severity (34). This view was not new as it had been the subject of discussions in the human field, where similar disparities in radiographic and clinical severity were indeed frustrating clinicians (35). This disparity has been explored through force-plate analysis which highlighted a poor correlation between radiographic OA and limb function (36, 37), as well as clinician- and owner-reported pain severity, which again were not associated with radiographic severity (38). Moreover, radiographic changes have been shown to be a poor predictor of cartilage damage when compared to arthroscopy (39). This poor correlation could be explained by the fact that radiography can only detect bony changes of osteophytosis and sclerosis, which only relate to a component of the joint organ disease and perhaps are not so well linked with drivers of clinical OA symptoms.

Radiography has restricted contrast resolution, with only five contrasts of opacity (air (gas), fat, water (soft tissue), bone (mineral) and metal). Radiopacity is determined by a tissue's inherent x-ray beam absorption and its thickness. The x-ray absorption of soft tissues is very similar to water, limiting the ability to differentiate between cartilage, synovial fluid and the soft tissue structures of the joint with plain radiography (40). The fidelity of radiography in OA diagnosis has been improved through the use of contrast arthrography to allow better visualization of structures, including articular cartilage, by temporarily changing the radiopacity of the surrounding joint fluid. In dogs, arthrography has been attempted particularly in the shoulder, and to a lesser extent the stifle, but with limited diagnostic value (41–43). So called advanced imaging modalities (CT and MRI) have made this approach somewhat redundant in plain film radiography, although ‘anesthetic arthrography', where contrast material is diluted with local anesthetic to identify both the source and cause of lameness has been used by some (44).

Despite its weaknesses, radiography remains the standard imaging modality for clinical diagnosis of OA in both humans and dogs (25, 45). While human and veterinary radiographic imaging of OA changes mostly align, there are some key differences with regard to subchondral bone cysts and joint space narrowing. Despite being described by Morgan (16) and being included in an extensive canine OA scoring system (46), subchondral bone cysts have not been as widely evaluated in dogs as in humans, where they are currently assessed as a key radiographic feature of OA (33, 47). Perhaps more striking is the discrepancy in the use of JSN; this is an indirect measure of human knee articular cartilage thickness in radiographs taken in a weight-bearing position (48). The importance of JSN in human medicine is highlighted through its selection as the recommended imaging end-point by the US Food and Drug Agency (FDA) in phase III clinical trials (31), although this currently being reviewed as shown by a recently published updated draft guidance (49). The application of JSN in veterinary medicine is, in contrast, very limited. Notwithstanding the need for horizontal beam radiography, which introduces additional radiation safety considerations, this raises questions about whether JSN assessment should be more widely used in veterinary medicine to improve the fidelity of this commonly available imaging modality.

Despite its limitations, radiography was a great leap forward in OA diagnosis in veterinary medicine during the 1970–late 1990s, allowing for internal appraisal of bone remodeling and some aspects of soft-tissue changes in OA, leading to earlier diagnosis of later stage OA pathology. However, the challenges of 2D superimposition, inability to assess cartilage directly and the insensitivity of radiography to different types of soft-tissue or fluid changes which may be present at earlier stages of OA were limiting.

## The Present and Future: Computed Tomography (CT)

The development of 3D X-ray imaging allowing planar segmental, and then multiplanar, and eventually 3D image reconstruction, was intimately linked to the development of computer micro processing power in the second half of the twentieth century. In 1971, a paradigm shift in medical and research imaging came with the first commercial CT scanner at the Atkinson Morley Hospital, London (50). The limitations of conspicuousness and subject contrast associated with 2D plain film radiography (51) and the potential solution to this had long been speculated with Radon describing the mathematical principles of tomography in 1917; sufficient computer power was not however available for a further 60 years. CT imaging uses mathematical algorithms to construct cross sectional images, based on a 2D matrix of pixels with their gray scale relating to different attenuation of x-rays through different tissues. These cross-sectional images are then reconstructed over the z-axis, providing the third dimension of the object (51, 52), thus removing the superimposition problem encountered with plain radiography. This gray scale of pixels can be converted into Hounsfield units (a quantitative scale for describing radiodensity), which using different processing algorithms can provide greater interrogation of various soft tissues for greater differentiation.

The capacity of CT imaging to differentiate superimposing structures has aided the ability to diagnose the bony changes picked up by plain film radiographs at an earlier stage, as complex joints can be interrogated in each plane. As scanner technology improved, this allowed for identification and quantitative assessment of even a single osteophyte and a degree of assessment of subchondral bone changes which would have not been visible in 2D plain radiography. Notably, although there are huge gains in spatial and intra-structural analysis, the actual resolution of a CT image remains lower than both digital and analogue radiography (53). Not surprisingly, the same hallmarks of OA are identified in CT as with radiography, as both techniques utilize x-ray radiation, although the Hounsfield scale allows for improved soft-tissue differentiation.

**Figure 5:**
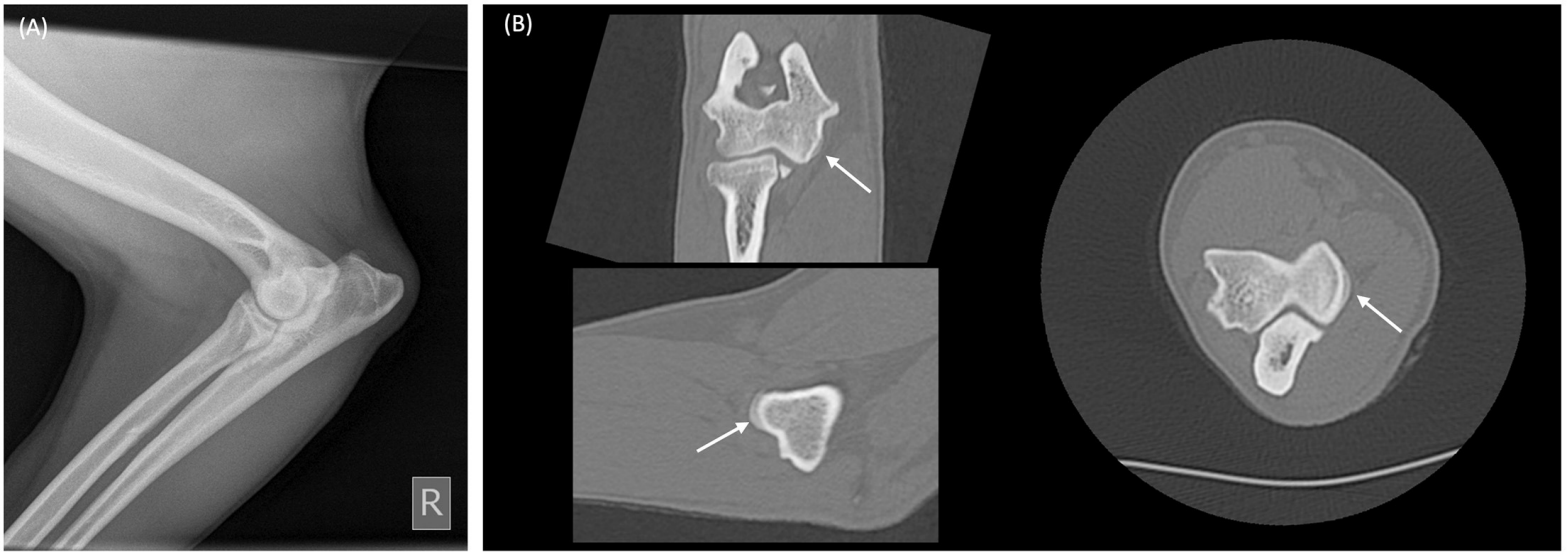
Radiograph and multiplanar reconstruction of a computer tomography (CT) of a canine elbow showing osteophytes are more easily identified on CT compared to radiography. **(A)** Mediolateral elbow radiograph from a skeletally mature dog showing minimal radiographic change of osteoarthritis. **(B)** Multiplanar CT reconstruction of the same elbow, demonstrates an occult 2 mm diameter osteophyte on the medial humeral condyle (white arrows).

The majority of the literature relating to musculoskeletal CT in dogs has focused on the elbow joint. The complex anatomy and the significant issue of superimposition at this site has led to CT being widely adopted by vets for assessment of the canine elbow with its superiority over conventional radiography being well-documented (54–56). It has also been recognized in human diagnostic imaging that CT is superior in detecting small osteophytes which are inconspicuous in plain 2D radiographs (57), and it can be assumed this applies to veterinary imaging (Figure 5). CT therefore allows earlier identification of the later secondary bony change while also allowing more accurate quantification of these measures. For example, osteophyte size can be measured by CT and has showed to be associated with articular cartilage changes confirmed arthroscopically within the elbow (58), with individual osteophyte size in CT imaging being the basis of most CT-based OA scoring systems in dogs (59). However, despite this increased sensitivity for bone remodeling, the articular cartilage remains invisible to CT without contrast-enhancement and the earlier, potentially reversible, OA changes still remain elusive.

Similar to radiography, the usefulness of contrast imaging with CT, CT arthrography (CTA), has been investigated in dogs (60–68), with CTA being able to delineate articular cartilage thickness indirectly as the measured structure between the subchondral bone and the contrasted enhanced synovial fluid, albeit prone to overestimated measurements (63). When compared, CTA can outperform CT in the assessment of cartilage shoulder joint lesions (64), however, CTA has not been widely adopted clinically for OA imaging, in part due to its perceived limited added value over CT and radiography.

**Figure 6:**
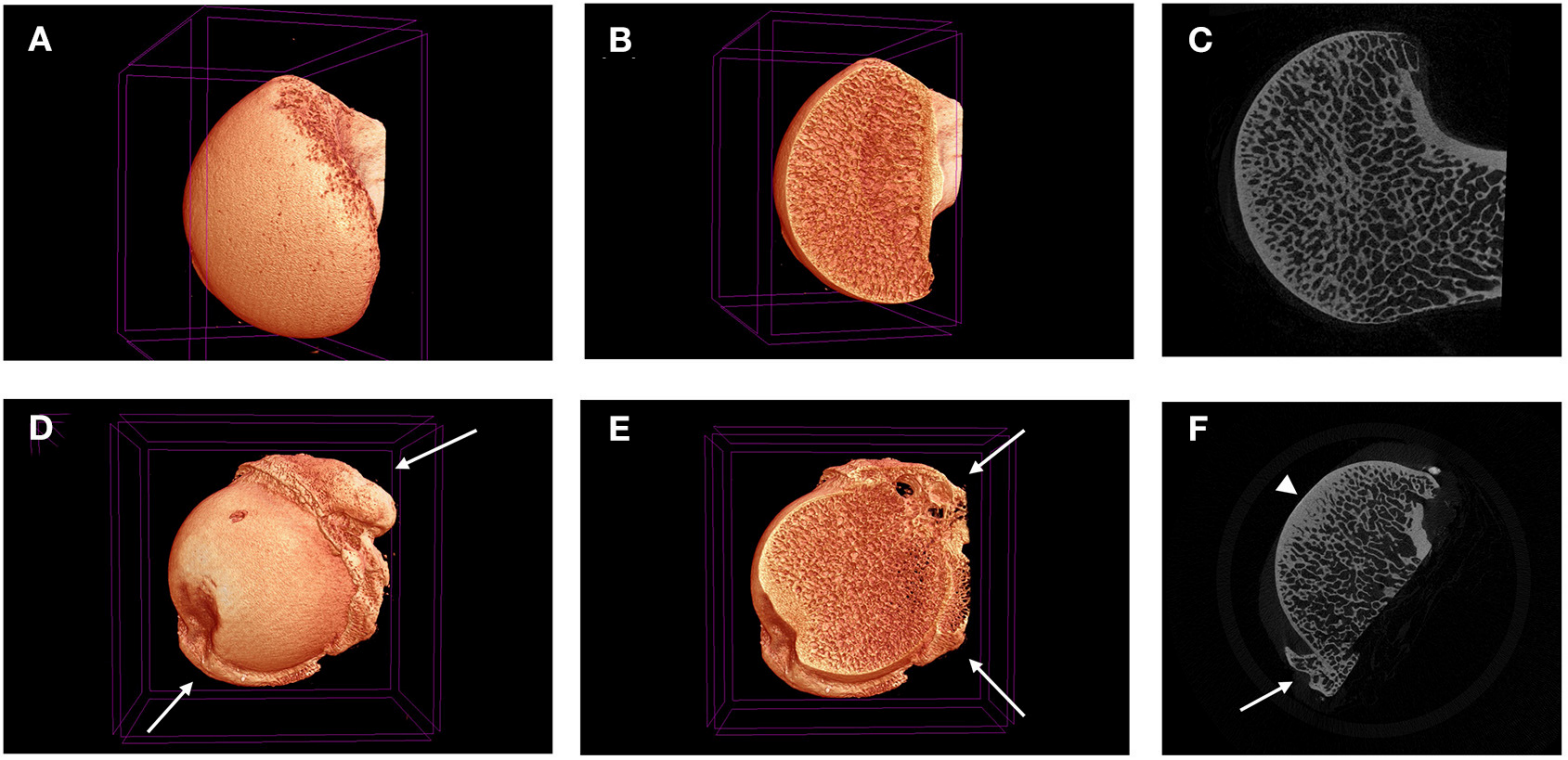
Micro-CT 3D reconstructions **(A,B,D,E)** and tonograms **(C,F)** of two canine femoral heads. Images **(A–C)** are taken from normal femoral head, while images **(D–F)** are taken from a diseased femoral head harvested during a total joint replacement. Peripheral osteophytes are visible in D-F (white arrows), as well as thickening in the subchondral bone plate (arrow head).

Other signs of OA have been identified in dogs using CT, including intraarticular gas (vacuum phantoms) (69), subchondral bone cysts in the elbow (55, 58, 59) and in degenerative sacroiliac joints (70). The search for predictive biomarkers of OA with CT has described several 2D and 3D measurements capable of identifying “at risk” dogs (71, 72), however, the identification of a significantly early imaging biomarker remains elusive. One potential area of interest however, is the assessment and quantification of subchondral OA bone changes, such as sclerosis, with CT. This sclerotic bone and its altered bone mineral density (BMD) has been assessed by CT in dogs (56, 73–75), with an increased BMD being explored as a quantifiable change associated with OA, in particular in the femoral head (76). Increased BMD was identified in both subchondral and non-subchondral bone in dogs with confirmed OA at post-mortem examination. This has been explored as a way to predict OA risk using young dogs as a model for human developmental hip dysplasia (77), although further research is required. Subchondral bone changes are promising, but CT probably does not have the resolution to interrogate these changes fully. However, the required level of resolution is commonplace in bone and joint research with extensive use of micro-CT (uCT) (78–84). Unlike clinical CT scanners which are commonly limited to 1–0.5 mm slices thickness, uCT allows assessment of bone at the microscopic level giving detailed assessment of OA associated bone changes at the trabecular structural unit level, with slices as thin as 5 μm (Figure 6).

Compared to simply looking at subchondral sclerosis or periarticular osteophytosis, a detailed assessment of subchondral bone structural change is expected to reveal earlier imaging biomarkers of OA development. Research is currently underway to better understand the interplay between cartilage and bone, and their respective changes as OA starts and progresses. It is envisioned that better quantification of microscopic level subchondral bone remodeling changes at different stages of cartilage degeneration, will translate into much earlier imaging biomarkers of disease. The current issue is the scalability as scanners cannot accommodate whole large animals and the radiation dose would be problematic. However, if the spatial relationships between cartilage degeneration and the underlying subchondral bone reported in humans (15) are seen in dogs, then identification of indicators of those uCT changes with clinical scanning technology may not only be possible, but may revolutionize OA diagnosis with earlier detection of previously unrecognized changes and thus setting an earlier limit of detection.

Access to CT imaging is improving in veterinary practice but remains limited, in part due to the high capital cost of the systems. Furthermore, its impact in OA imaging is limited by its insensitivity in detecting cartilage lesions and ultimately that it does not currently allow for significantly earlier detection of OA. Moreover, the increased radiation dose compared to radiography should be considered, especially where repeated imaging is being performed. With all of this combined, the search for earlier detection of OA, while it is still potentially reversible, has expanded beyond X-ray based technology.

## The Present and Future: Magnetic Resonance Imaging

A second paradigm shift in OA imaging occurred with the invention of MRI, which for the first time allowed detailed imaging of soft-tissues and bone, giving holistic interrogation of the whole joint organ. MRI exploits the electromagnetic properties of hydrogen ions in H_2_O and lipids, which are abundantly present in tissues throughout the body, by application of intense magnetic fields to the tissues and then measuring their differential response as radiofrequency emissions (85). Depending on the sequences involved, different aspects of the joint organ can be highlighted in any particular scan by utilizing the specific T1 relaxation (the time taken for the spinning protons to align themselves to the external magnet) and T2 relaxation (the time taken for the transverse magnetization vector to decay due to dephasing of the spinning protons) times of each tissue within the joint. Relaxation time refers to the time taken for an excited molecule to return to its equilibrium state. The common sequences currently used in musculoskeletal MRI include T1-weighted and T2-weighted echo sequences, fat suppression sequences (86), as well as short tau inversion recovery (STIR) and proton density sequences among others (87). MRI has become the gold standard for joint imaging in humans as it allows multiplanar imaging of the whole joint organ, with excellent soft tissue and good bone definition.

In dogs, MRI has been widely adopted for neurological assessment, but not for OA. One of the first publications referencing the use of MRI to assess canine experimental OA was nevertheless published as far back as 1987 (88), in which changes of the stifle in a cranial cruciate ligament (CCL) transection model were evaluated. The authors showed that MRI changes correlated to gross pathology and that the changes linked to OA were seen on MRI earlier than with radiography. In this study, osteophytes were visible on MRI 4 weeks after CCL transection while they were not visible on radiography until 12 weeks post transection (88). The lack of uptake of MRI in veterinary clinical practice for joint assessment is in large part due to it having to compete with modalities such as plain radiographs and CT that are well established and give high detail of bony change in OA at a fraction of the cost. MRI has several drawbacks for a perceived limited gain, including limited access to sufficiently powered scanners, long sequence acquisition times compared to other imaging modalities, especially as general anesthesia is required to reduce movement artifact. Of all the imaging modalities discussed, this is where the greatest difference between the imaging of OA in veterinary medicine and human medicine lies. The majority of MRI reports in the dog have focused on experimental canine models of human knee OA, which have been used since the 1970's (89). Clinically, the use of MRI in dogs has focused mainly on the shoulder, as it allows excellent assessment of both intra- and extra-articular structures, and in particular the musculotendinous structures commonly causing shoulder lameness in the dog (87), and has not been explicitly explored for shoulder OA.

**Figure 7:**
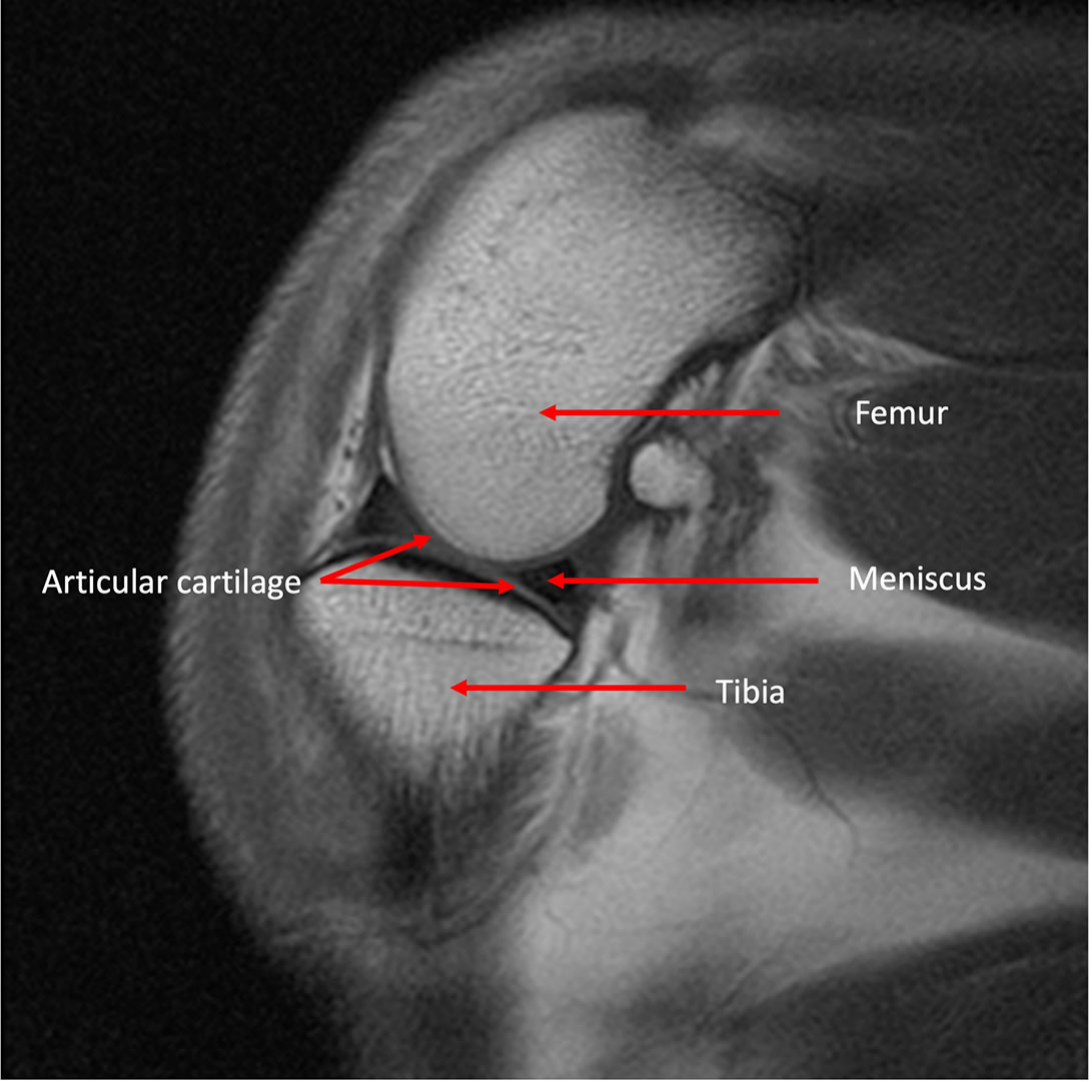
MRI T1 weighted sagittal image of a canine stifle identifying both bone and soft tissue anatomy.

Unlike radiography and CT, MRI was the first imaging modality to be able to directly image cartilage and to have a good level of resolution for soft as well has hard joint tissues (Figure 7). Experimentally, it has been demonstrated to be a more sensitive imaging modality in identifying the onset and progression of OA in dogs, in particular with osteophytosis (90). However, the direct assessment of articular cartilage is quite unique. This is possible due to the significant water content of cartilage (~80%). Several MRI studies using experimental dog OA models have shown that it is not only possible to measure thickness of articular cartilage, but also to assess lesion progression *in-vivo* (91–93). Interestingly, being able to follow dogs with CCL transection over an extended period documented that cartilage hypertrophy in response to joint destabilization can persist for up to 3 years, before the cartilage shows evidence of thinning (91, 93). However, the visualization of cartilage is dependent on the MRI equipment and protocols applied, as when larger slice thicknesses are used (such as 1 cm) and a low field system, the ability to detect cartilage lesions is lost (88). This is in part related to the relative cartilage thickness in canine joints being thinner than in human joints, which necessitates the use of higher MRI field strength magnets and thinner slice thicknesses. Nevertheless, MRI has opened the door to allow direct assessment of cartilage, a key aspect of the osteoarthritic joint previously not accessible non-invasively.

**Figure 8:**
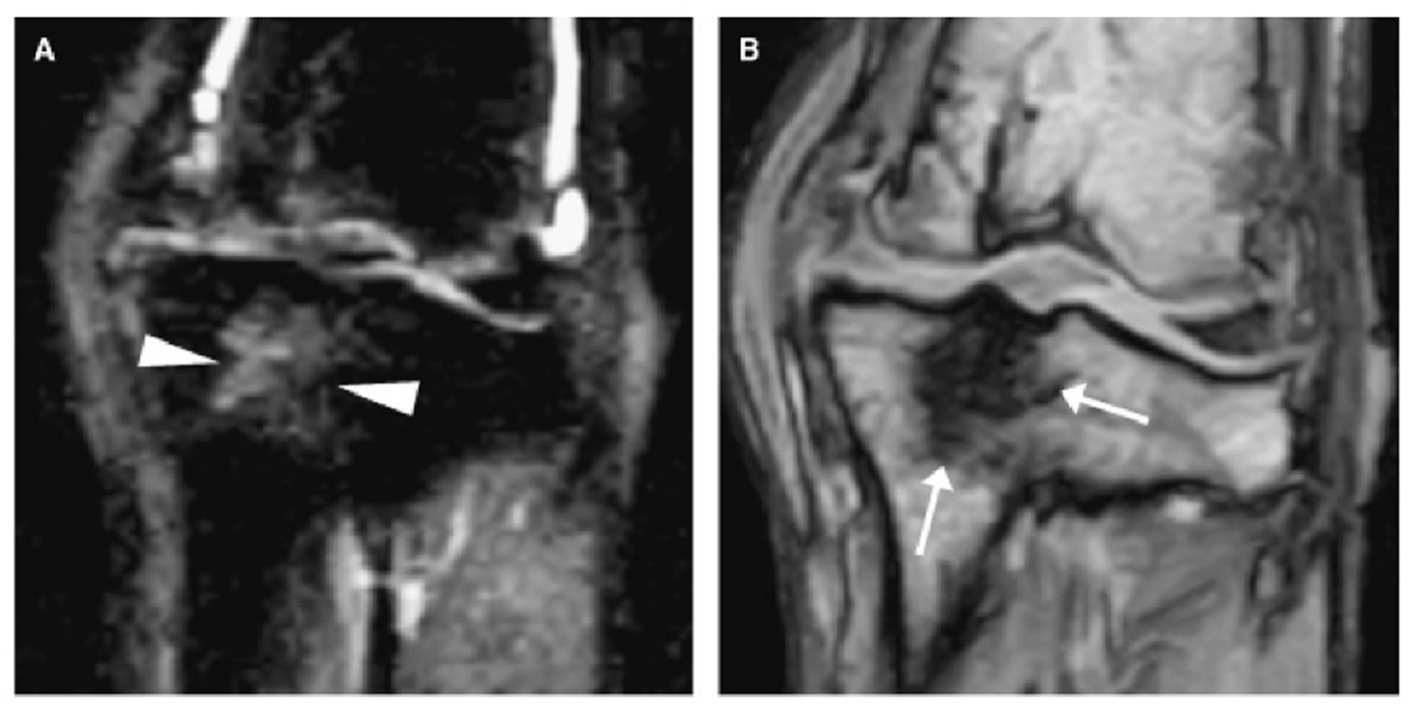
Two dorsal stifle MRI images of a dog 13 months after a CCL transection showing a bone marrow lesion (BML) within the dorsal tibia. **(A)** is a short tau inversion recovery sequence (STIR) which shows the BML as a heterogeneous hyperintensity in the dorsal tibia (arrow heads), while **(B)** is a T1-weighted sequence where the BML appears as a hypointense lesion (arrows). MRI – Magnetic resonance imaging; CCL – cranial cruciate ligament. Martig S et al. MRI characteristics and histology of bone marrow lesions in dogs with experimentally induced osteoarthritis. Vet Radiol Ultrasound. 2007; 48(2):105–12. Reprinted with permission.

Promisingly, hitherto unknown and early changes associated with OA were revealed when this approach was applied to OA joints. So called BMLs were identified as hyperintense regions on T2 weighted, fat suppressed sequences in human OA joints (14). The underlying aetiopathogenesis of these lesions is not well understood as histological assessment in human patients is limited. Their presence within the epiphyseal trabecular bone made them elusive to 2D imaging as they were effaced by overlying structures, and their soft-tissue/fluid nature made them invisible to X-ray beam differentiation. Canine experimental models suggest these BMLs represent fat necrosis and/or fibrosis within the bone (94, 95). These lesions have attracted attention as both a possible early reversible biomarker and a source of pain in OA (96). While most of the research into BMLs has focused on human OA, their presence in dog OA has been explored in canine experimental models, with BMLs being identified in dogs as early as 4–6 weeks post stifle destabilization (95, 97–99) (Figure 8). There are also reports of BMLs in dogs with spontaneous stifle and elbow disease (100–102), and hence they may be a viable earlier clinical indicator of OA pathology. The discovery of BML with MRI nicely highlights that with the correct imaging modality, earlier or previously unknown imaging biomarkers may become identifiable in OA as new technologies develop. This is further highlighted as other imaging biomarkers have also been identified with the use of MRI, notably the identification of high density mineralised protrusions (HDMPs) from the calcified cartilage into the hyaline cartilage in both equine and human OA (103, 104).

As a non-invasive modality which can directly assess bone and soft-tissue joint components and in particular articular cartilage, MRI is commonly used in human phase III clinical trials leading to the development of several semi-quantitative scoring systems, such as the Whole-Organ Magnetic Resonance Imaging Score (WORMS) (86) among others (31). These scoring systems assess the whole organ, unlike the radiographic systems, making them well suited for both cross-sectional and longitudinal studies. It is likely that the sum total of many very minor changes particularly in the earlier disease stages may facilitate confident prediction of later OA. Currently, there are no semi-quantitative scoring systems available in dogs.

In human medicine clinical research MRI scanners of increasing resolution combined with new protocols are starting to allow interrogation of the composition of the joint, including makers of glycosaminoglycan (GAG) content and articular cartilage structure. Inevitably, this will reveal earlier OA disease states, or new previously hidden aspects. In time this technology will be shared with veterinary patients as well.

## Nuclear Medicine and Others

The mainstream of imaging modalities in OA reveal structural change, however some of the earliest stages of OA development include metabolic rather than gross structural aberrations. Currently only used in research or in select non-OA clinical conditions, gamma scintigraphy, positron emission and thermography could thus provide new strategies to look further back to the earliest stages of OA.

Gamma scintigraphy is a form of nuclear medicine, which utilizes the gamma radiation emitted from the administration of radioactive isotopes, primarily Technetium-99m (^99m^Tc), which can be bound to specific tissue markers. Phosphate bound ^99m^Tc is preferentially absorbed by bone at locations of greater osteoclast: osteoblast mediated (re)modeling, which is identified as a ‘hot spot' when scanned with a gamma-camera (105). Intriguingly, it has been shown that there is greater uptake of ^99m^Tc-labeled markers specifically in joints with OA (106, 107) with defined abnormal early and late phase isotope patterns (108). These isotope patterns are thought to represent different aspects of OA; the early phase pattern identifying synovitis and the late phase pattern identifying osteophyte formation. This approach, although sensitive to early OA disease is currently hampered by poor spatial resolution and poor specificity for the underlying pathology, along with the inherent difficulties associated with managing radiopharmaceuticals. In the veterinary clinic its main application has been to identify a hitherto unidentifiable source of lameness which can then be further interrogated with more conventional investigations (109). Considering its targeting mechanism is aligned to any particular tissues metabolites being radioactively tagged, new targeted markers, such as ^99m^Tc labeled chondroitin sulfate which is selectively taken up in dogs with cartilage degeneration (110), may open up further avenues for scintigraphy, especially with the improved spatial resolution with single proton emission computed tomography (SPECT/CT).

**Figure 9:**
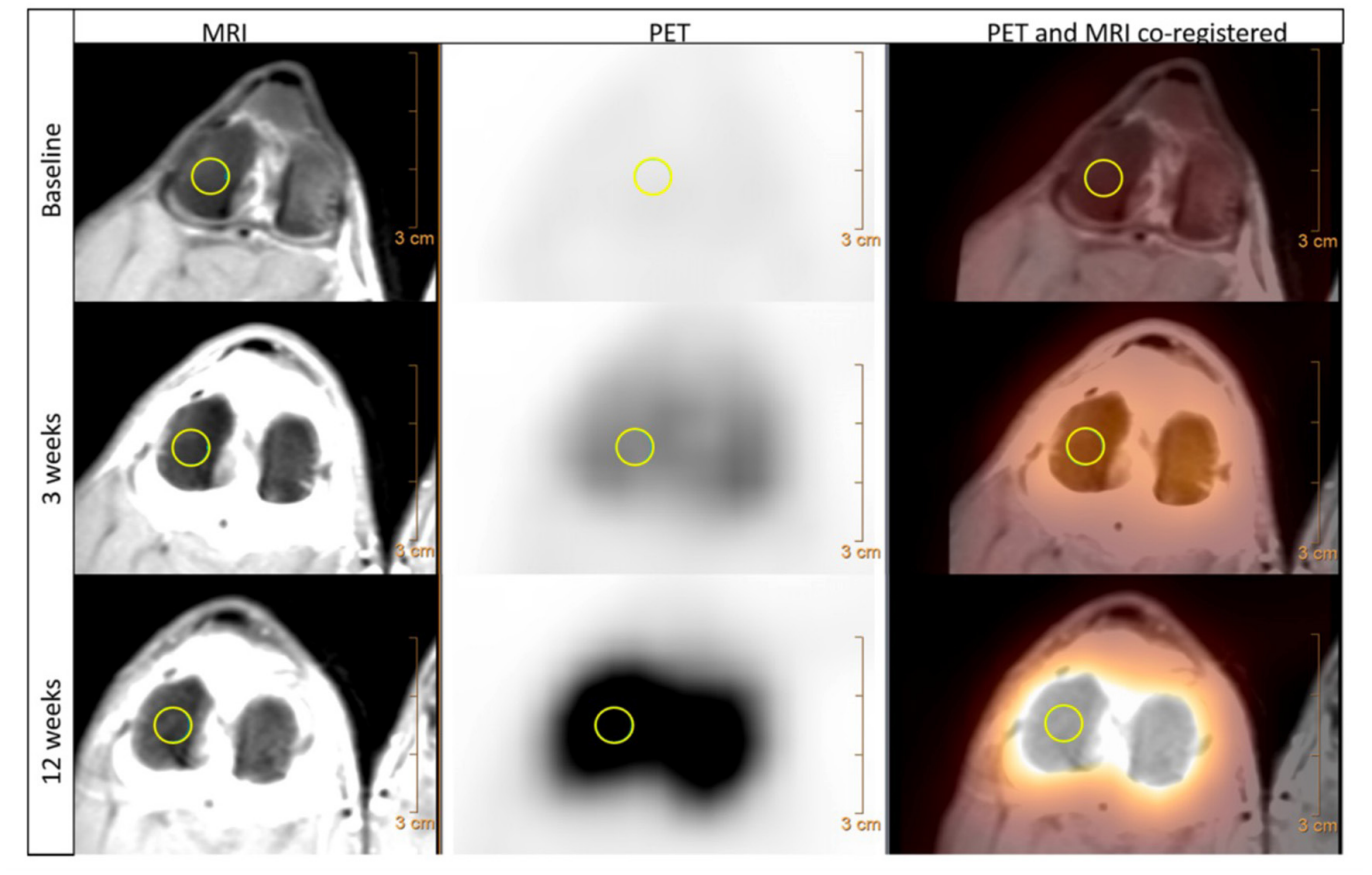
MRI, PET and PET/MRI co-registered transverse views of a canine stifle using Na18F before (baseline), 3 weeks and 12 weeks following CCL transection. The MRI images used a proton density weighted sequence and depict the detailed anatomy of the stifle. The Na18F PET imaging shows an increase of positron emissions compared to baseline at both 3 weeks and then again at 12 weeks post CCL transection, indicating an increase bone uptake of Na18F in response to increase bone metabolism following joint destabilization. Both images are co-registered allowing direct assessment of both the anatomic and metabolic changes, with a manually drawn 6 mm 3D region of interest present on the lateral femoral condyle (yellow circle). MRI – Magnetic resonance imaging; PET – positron emission tomography; CCL – cranial cruciate ligament. Menendez M et al. Feasibility of Na18F PET/CT and MRI for Noninvasive *In vivo* Quantification of Knee Pathophysiological Bone Metabolism in a Canine Model of Post-traumatic Osteoarthritis. Mol Imaging. 2017: 16 (1–8); 10.1177/1536012117714575. Reprinted under creative common license.

Positron emission tomography (PET) is another form of nuclear medicine, which allows functional imaging of tissue metabolism. Recent technological advancements have led to combined PET/CT and PET/MRI machines, improving the spatial resolution by allowing the combination (co-registration) of both the metabolic and anatomic scans. Previously, patients would need to be scanned in two machines and a complex process of image registration (overlaying one image on top of another) would be required. PET imaging utilizes positron emitting radiopharmaceuticals such as Fluorine-18 (^18^F) instead of gamma emitting compounds. Use in veterinary medicine has primarily been based around oncology (111), although it has been explored as an adjuvant for soft tissue lameness investigation by using fluoro-2-deoxy-D-glucose (^18^F-FDG), a glucose analogue (112). In relation to bone metabolism, sodium fluoride-^18^F (^18^F-NaF) has also been shown to be a feasible bone metabolic marker in an experimental canine OA model, showing increased uptake in the operated limb at 3 weeks and 12 weeks post-surgery, highlighting its potential as an early OA biomarker (113) (Figure 9). The use of ^18^F-NaF in the detection of early OA is being explored in human OA imaging, with some early results suggesting it might even be able to detect OA prior to visible early morphological changes (114). While outside its use as an adjuvant imaging modality for difficult-to-locate lameness or the research setting, it is challenging to see this modality being integrated into routine clinical practice. This is further compounded by the challenges of sourcing, storage and using of radiopharmaceuticals in veterinary practice.

Another non-invasive diagnostic modality that has been investigated is the use of thermography. It has been used in dogs to help locate the site of OA by measuring body surface temperature (115) and was found to be capable of differentiating between normal and diseased joints (116, 117). More recently, its use in OA identification within a working dog population has been explored (118, 119). However, body surface temperature can be affected by hair coat characteristics (120) and by coat clipping (121), which can complicate the interpretation of the thermograms. Moreover, similar to other ancillary methods, it is limited by equipment cost and its poor specificity, with further research in dogs required to determine whether it identifies OA prior to radiographic later changes.

## The Future: What Could be Next?

**Figure 10:**
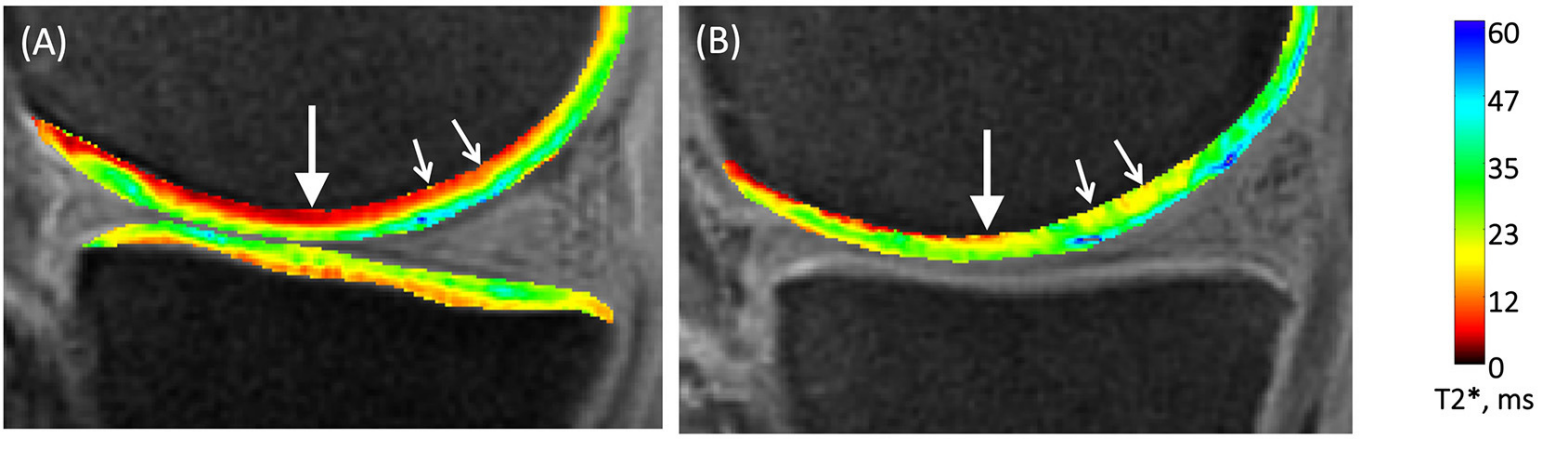
Compositional MRI of two human knees using UTE-T2* mapping. **(A)** UTE-T2* map of an un-injured human knee shows a lower signal (red) in the deeper layers of the articular cartilage (arrows). **(B)** UTE-T2* map of a knee acutely following an ACL-injury, shows a higher more disorganized signal pattern in the deeper layers of the articular cartilage. MRI – Magnetic resonance imaging; UTE – ultrasound time echo; ACL – anterior cruciate ligament. Chu C et al. Visualizing Pre-osteoarthritis: Integrating MRI UTE-T2* with mechanics and biology to combat osteoarthritis—The 2019 Elizabeth Winston Lanier Kappa Delta Award. Journal of Orthopedic Research. 2021; (1–11). Reprinted with permission.

**Figure 11:**
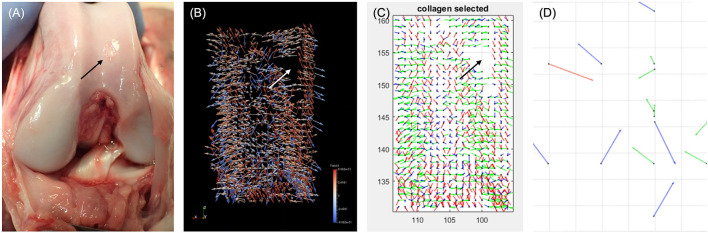
Photograph of a canine stifle at *post mortem* with the associated collagen tractography using Magic Angle Directional MRI. **(A)** Photography of canine stifle at *post mortem* showing a defect in the cartilage (black arrow). **(B)** Coronal ParaView glyph vector visualization of the collagen fiber orientation following Magic Angle Directional MRI of the femoral condyles showing a signal void (white arrow), consistent with the cartilage defect. **(C)** Fiber orientation map showing the tractography of the collagen fibers from the same canine stifle, again identifying a signal void (black arrow). **(D)** Fiber orientation map focusing on the area of signal void, identifying disorganized fiber orientation. Chappell K. Optimisation and visualization of collagen fiber orientation using Magic Angle Directional Imaging (MADI). [PhD Thesis]. 2019. Imperial College London, UK. Reprinted with permission from Dr Karyn Chappell.

This review has demonstrated a clear relationship between the development of technology from radiography in the 1960s, CT in the 1970s, MRI in the 1980s, with the associated expansion and development of computing power, and the pushing-back of the limits of OA detection to progressively earlier stages. It is therefore reasonable to speculate–and there are promising signs–that this will progress to the point where OA is detectable at stages when it is reversible. A hint of this capacity is shown with compositional MRI which exceeds current capabilities by facilitating assessment of cartilage topography and hydration status. Compositional MRI techniques gives an indication of the biochemical/compositional changes developing in cartilage, potentially at the earliest stages of OA. Several different techniques exist, which allow evaluation of different aspects of cartilage; from the collagen network, its water content or its GAG concentration. This is of particular interest, as GAG content changes occur very early in the aetiopathogenesis of OA (10, 78). These composition techniques have been used in human research over the past two decades (31), with some of them being translated into the dog. Preliminary studies have been performed in dogs using delayed gadolinium-enhanced MRI of cartilage (dGEMRIC) and T2 mapping to identify normal reference ranges in the canine elbow joints (122) and for the normal stifle (123). Furthermore, there has been a comparison between delayed intravenous (dGEMRIC) and intraarticular (iGEMRIC) administration of gadolinium contrast mediums in dogs (124). However, in a population of dogs with an induced osteochondral defect, there was only a weak T1p imaging correlation with cartilage change and no correlation with T2 mapping (84). Combined with quantitative MRI techniques, it is conceivable that this could replace arthroscopy in certain circumstances. Moreover, recent work in human medicine with a new compositional MRI sequence, ultrashort echo time enhanced (UTE) T2^*^ mapping, is driving a paradigm shift toward identifying and mapping the “Pre-osteoarthritic” joint (125) (Figure 10). The hope is that these “pre-OA” biomarkers represent the ever-elusive biomarkers of early disease prior to irreversible joint demise, and will fuel development of interventions which are able to reverse OA progression. A further emerging area with MRI imaging, is using magic angle directional imaging to identify the dominant collagen fiber orientation (126). This application of MRI allows mapping of collagen bundles within soft-tissues which could provide other indicators of the structural derangements occurring in the cartilage (Figure 11).

## A Second Look?

It is also possible that rather than looking to new or improved imaging modalities, the current ones may already be able to detect the earlier changes in OA, but we simply do not know what those subtle changes or combination of changes are. This prospect could be realized through the application of machine learning and deep learning, which have already been shown to be able to aid imaging segmentation, disease diagnosis and image reconstruction (127–129). Both machine learning and deep learning are branches of artificial intelligence (AI), which have expanded exponentially over the past decade. One example of the application of deep learning in medical imaging, is its ability to predict patients who required total knee replacements due to OA via standard radiographs (130). Machine and deep learning have already been applied to some areas of veterinary medicine including thoracic radiographs (131) and intracranial neoplasia differentiation (132). It seems likely that these AI methods will have the potential not only to improve OA imaging diagnosis but to identify predictive biomarkers of which we were hitherto unaware.

## Summary

Since Morgan's publication in 1969 outlining the radiographic changes in the dog, the imaging of OA in dogs has advanced beyond recognition. Each advancement in imaging technology and development of resolution has driven back the point of identification of OA to an earlier stage of gross structural disease. With the increasing access to high-field MRI systems within veterinary practice, consideration of collagen structure, metabolic changes and increased fidelity of imaging modality and interpretation with AI, it is likely that the biochemical changes which precede structural markers will be identifiable through imaging. There is clear promise to moving the limit of detection of OA to the early and hopefully reversible metabolic changes which could conceivably usher in a paradigm shift in OA management.

## Author Contributions

GJ, AP, and RM contributed to conception, design of the study, and discussions of content. GJ researched data for the article and wrote the first draft of the manuscript. All authors contributed to manuscript revision, read, and approved the submitted version.

## Conflict of Interest

The authors declare that the research was conducted in the absence of any commercial or financial relationships that could be construed as a potential conflict of interest.

## Publisher's Note

All claims expressed in this article are solely those of the authors and do not necessarily represent those of their affiliated organizations, or those of the publisher, the editors and the reviewers. Any product that may be evaluated in this article, or claim that may be made by its manufacturer, is not guaranteed or endorsed by the publisher.

## Abbreviations

AI: Artificial intelligence
BMD: Bone mineral density
BML: Bone marrow lesion
CCL: Cranial cruciate ligament
CT: Computed tomography
CTA: Competed tomography arthrography
FDA: US Food and Drug Agency
FDG: Fluoro-2-deoxy-D-glucose
GAG: Glycosaminoglycans
HDMPs: High density mineralised protrusions
KL: Kellgren-Lawrence
JSN: Joint space narrowing
MRI: Magnetic resonance imaging
OA: Osteoarthritis
PET: Positron emission tomography
Tc: Technetium
uCT: Micro-computed tomography
UTE: Ultrashort echo time enhanced.
